# Which therapeutic exercise is most effective for improving ankle inversion muscle function in individuals with chronic ankle instability? A systematic review and network meta-analysis

**DOI:** 10.3389/fbioe.2025.1691203

**Published:** 2025-12-18

**Authors:** Haipeng Zhang, Lijiang Luan, Xiaojian Shi, Jingwang Tan, Roger Adams, Hong Wang, Jia Han

**Affiliations:** 1 Department of Rehabilitation, JiangWan Hospital of Hongkou District, Shanghai University of Medicine and Health Sciences, Shanghai, China; 2 School of Exercise and Health, Shanghai University of Sport, Yangpu, Shanghai, China; 3 School of Physical Education and Health, Sanming University, Sanming, Fujian, China; 4 School of Health and Biomedical Sciences, Royal Melbourne Institute of Technology University, Bundoora, VIC, Australia; 5 College of Physical Education, Shanghai University, Shanghai, China; 6 Research Institute of Sport and Exercise, University of Canberra, Canberra, ACT, Australia; 7 College of Rehabilitation Sciences, Shanghai University of Medicine and Health Sciences, Shanghai, China

**Keywords:** chronic ankle instability, therapeutic exercise, ankle inversion muscle, neuromuscular training, strength training, combined neuromuscular and strength training, combined neuromuscular and whole-body vibration training

## Abstract

**Objective:**

This study was conducted to investigate the effects of diverse therapeutic exercise regimens on enhancing ankle inversion muscle function in individuals with chronic ankle instability (CAI).

**Methods:**

A systematic search was performed across five international electronic databases (e.g., PubMed, Cochrane Library, Embase, Web of Science, and EBSCO) from their inception to December 2024. Randomized controlled trials focusing on the keywords “ankle instability,” “exercise therapy,” and “muscle strength” was screened. Data extraction and quantitative analysis were subsequently executed. Methodological quality and risk of bias were assessed with Cochrane Collaboration Bias Risk Assessment Tool.

**Results:**

Nine studies involving 366 participants were included in the network meta-analysis (NMA). The findings demonstrated that all therapeutic exercise modalities (neuromuscular training (NT), (Effect size (ES) = 1.05; 95%CI = 0.29–1.82), strength training (ST), (ES = 1.36; 95%CI = 0.73–1.99), combined neuromuscular and strength training (NST), (ES = 2.82; 95%CI = 1.89–3.74) and combined neuromuscular and whole-body vibration training (NWBVT), (ES = 1.92; 95%CI = 0.62–3.22) exhibited statistically superior efficacy compared to control groups. Surface Under the Cumulative Ranking Curve (SUCRA) probability rankings identified NST as the intervention with the highest likelihood of optimal efficacy (99.9%). ST (64.6%) alone demonstrated a significant advantage over NT alone (45.9%). Conversely, NWBVT was associated with the lowest therapeutic probability (38.9%).

**Conclusion:**

Combined neuromuscular and strength training (NST) constitutes the most effective therapeutic exercise for augmenting ankle inversion muscle function in CAI populations. Compared to isolated NT or ST interventions, the integration of these modalities demonstrates greater efficacy in addressing functional impairments in CAI individuals.

## 1 Introduction

Sport injuries are widely recognized as a substantial global health burden, with restrictions on participation and considerable economic costs (Turnbull et al., 2024; Lutter et al., 2022). Lower-limb injuries are strongly associated with impaired performance and reduced quality of life (Sleeswijk Visser et al., 2021). The ankle joint function is critical for weight-bearing, postural stability, and rapid directional changes in daily and athletic activities (Wellborn et al., 2023). Ankle sprains have been consistently documented as a predominant sports-related low limb injuries, accounting for approximately 10%–30% of all sports-related injuries (Song et al., 2019). Poor management of this condition may lead to long-term dysfunction (Doherty et al., 2014; Thompson et al., 2018). A history of ankle sprains has been reported in approximately 46% of individuals progressing to chronic ankle instability (CAI) (Lin et al., 2021). CAI is characterized by recurrent ankle sprains, restricted joint mobility, and neuromuscular control deficits in balance and postural stability, associated with structural and/or functional impairments in the ankle joint and surrounding tissues (Delahunt et al., 2010).

Ankle inversion muscle function is defined as the muscular capacity needed to maintain normal inversion movement of the ankle joint under external forces. This is characterized by the maximal force that can be generated in the inversion direction, as measured by an isokinetic dynamometer. It has been demonstrated that individuals with CAI are consistently associated with diminished ankle inversion strength (Khalaj et al., 2020; Peng et al., 2024). This deficit has been identified as a primary contributor to the functional and perceived instability of the ankle joint observed in CAI individuals (Peng et al., 2024). The tibialis anterior, tibialis posterior, and extensor hallucis longus are identified as the primary muscles responsible for ankle inversion (Shah et al., 2025). Kinesiological and biomechanical evidence has demonstrated that the tibialis posterior and tibialis anterior contribute substantially to inversion torque and mediolateral joint control, thereby reducing excessive inversion moments and enhancing joint stability (Maharaj et al., 2016; Semple et al., 2009; Shah et al., 2025). The tibialis posterior has been described as a dynamic stabilizer during the stance phase, resisting pronatory forces and maintaining alignment (Maharaj et al., 2016; Semple et al., 2009). The tibialis anterior provides an additional inversion component during closed-chain tasks, facilitating modulation of frontal-plane foot motion (Shah et al., 2025; Zhang and Sun, 2024). Overall, dysfunction or weakness of these invertor muscles has been associated with diminished counter-torque capacity, altered kinematic responses under perturbation, leading to increased reliance on passive stabilizing structures (Simpson et al., 2019; Komatsu et al., 2024). Moreover, evidence suggests that inversion function deficits in CAI individuals may arise from arthrogenic muscle inhibition, a selective neuromuscular suppression mechanism (Ryan, 1994). Specifically, following lateral ankle sprains, a sustained reflexive response is triggered in periarticular tissues, manifesting as inhibition of muscular activation to avoid irritating the lateral ankle regions (e.g., lateral ligamentous tissues). However, the prolongation of such protective inhibition can impede long-term functional rehabilitation (Hopkins and Ingersoll, 2000). During sudden inversion stress, insufficient activation of inversion-controlling musculature (e.g., tibialis posterior, tibialis anterior) results in disproportionate stress transfer to ligamentous structures, compromising dynamic joint stability. Therefore, targeted strengthening of the ankle inversion muscle function is considered a necessary intervention for the enhancement of ankle joint stability (Wang et al., 2022; Sekir et al., 2007; Park et al., 2024; Park et al., 2021; Hall et al., 2015; Smith et al., 2012). In accordance with this mechanism, some studies have investigated the effect of ankle inversion muscle strengthening exercise on ankle stability and self-reported outcomes. A recent study indicated that a 6-week isokinetic strength training intervention significantly improved ankle inversion muscle strength, dynamic balance ability (Star Excursion Balance Test-SEBT) and self-reported outcome (Cumberland ankle instability tool-CAIT) in participants with ankle instability (Wang et al., 2022). This evidence has highlighted the importance of effective therapeutic exercise selection strategies, as it established a direct link between muscle function and clinical outcomes.

A wide range of therapeutic training protocols have been implemented in individuals with CAI, which can be broadly categorized into proprioceptive training and strength training (Zhang et al., 2025; Antohe and Panaet, 2024). However, these protocols generally aim to improve overall function in CAI individuals without targeting specific aspects of lower limb function. Recently, a number of studies have used neuromuscular training (NT), isometric or isotonic strength training for the ankle joint, and combined exercise programs to address ankle inversion muscle dysfunction in CAI (de Vries et al., 2011; Cruz et al., 2019; Bagherian et al., 2019; Shamseddini Sofla et al., 2021).

These findings highlight the importance of investor strengthening in CAI, yet the most effective therapeutic exercise is unknown. Clinical studies report inconsistent outcomes for the various exercise interventions that have been used to enhance ankle inversion muscle function, necessitating resolution through network meta-analysis (NMA) (Cruz et al., 2019; Hall et al., 2015; Hall et al., 2018; McKeon et al., 2008). Accordingly, this NMA aimed to evaluate the efficacy of diverse exercise therapies on ankle inversion muscle function in CAI individuals, via examination of relevant randomized controlled trials (RCTs), which may inform optimal interventions selection in clinical settings.

## 2 Methods

This systematic review and NMA were conducted according to the Preferred Reporting Items for Systematic Reviews and Meta-Analyses (PRISMA) statement guideline. Preferred Reporting Items for Systematic Reviews Incorporating Network Meta-Analyses (PRISMA Extension Statement) guidelines and Systematic Reviews and Meta-Analyses (PRISMA 2020 statement) guidelines (Hutton et al., 2015). The study protocol was registered in the International Prospective Register of Systematic Reviews (PROSPERO) on February 4th, 2025 (CRD42025646752).

### 2.1 Data sources and searches

Databases searched included PubMed, Cochrane Library, Embase, Web of Science, and EBSCO, all performed in December 2024, restricted to English-language publications and no restriction of publication year. The search strategy used a mix of Mesh and free text terms from the following group of 4.1. “Joint Instability [Mesh]”, “Joint Instabilities”, “Joint Hypermobilities”, “Joint Hypermobility”, “Joint Laxities”, “Joint Laxity”. 2. “Ankle Joint [Mesh]”, “Ankle Joints”, “Articulatio talocruralis”, “Ankle Syndesmos*”, “Talocrural Joint*”, “Tibiofibular Ankle Syndesmos*”, “Distal Tibiofibular Joint*”, “Inferior Tibiofibular Joint*”, “Tibiofibular Syndesmos*”. 3. “Exercise [Mesh])”, “Exercises”, “training”, “Aerobic Exercise*”, “Aerobic training”, “Isometric Exercise*”, “Exercise Training*”, “moderate intensity continuous training”, “resistance training”, “strength training”, “combined training”, “sprint interval training”, “high intensity interval training”, “neuromuscular training”. 4. “randomized controlled trial”, “randomized”, “placebo”. Each keyword and Mesh (or Publication Type) in a group were combined with an “OR” operator, and the operation of “AND” was used for merging between groups.

### 2.2 Study selection

Two authors (H.P.Z. and X.J.S.) independently screened the titles and abstracts of potentially eligible studies and retrieved the full texts of the remaining articles to assess their eligibility based on keywords relating to the PICOS tool. The keywords were: (a) population: individuals with CAI; (b) intervention: therapeutic exercise; (c) comparison: no intervention, a placebo, or an alternative therapeutic exercise different from experimental group; (d) outcome: ankle inversion muscle function; (e) study design: RCT. Any discrepancies between the 2 authors were resolved by discussion and consultation with other members of the review team (L.J.L.).

Studies were considered eligible for inclusion if the following criteria were met: (a) individuals diagnosed as CAI, regardless of age or sex; (b) RCT. (c) the experimental group received therapeutic exercise, either as a stand-alone intervention or as part of a multimodal program, while the control group was administered either no intervention, a placebo, or an alternative therapeutic exercise different from that administered to the intervention group. (d) outcome measures included ankle inversion muscle function. The following were excluded: (a) if the characteristics of control group were different from that of experimental group. (b) if the observation of the control and experimental conditions conducted on the unaffected and affected ankles of the same participants. (c) if the articles were reviews, clinical registrations, meeting abstracts, case reports or full texts not available.

### 2.3 Quality assessment

The risk of bias from individual studies was independently assessed by two investigators (H.P.Z. and X.J.S.) in accordance with the Cochrane Handbook for Systematic Reviews of Interventions Version 7.2.0 tool for assessing risk of bias in RCTs (Sterne et al., 2019). The overall risk of bias judgment for each included study was calculated considering the following domains: (a) randomization process; (b) measurement of the outcome; (c) missing outcome data; (d) selection of the reported result; and (e) other bias. Eligible studies were classified into 3 levels of risk of bias (e.g., high, moderate, and low) by the number of domains for which high, unclear, or low risk of bias potentially existed.

### 2.4 Data extraction

All data were extracted from the included studies by 2 investigators (H.P.Z. and X.J.S.) using standardized data extraction forms. The extracted data information included the author of the study, the year of publication, the demographical characteristics of the participants (total number and gender ratio, mean age, height and weight), exercise intervention details (type, frequency, duration) and outcome measures reported from each eligible study as shown in table. The measurement of the outcome indicators of ankle inversion function was conducted using specialized equipment for measuring muscle function. The testing instruments mainly consisted of isokinetic force-measuring devices such as Biodex, the SYSTEM 3 PRO dynamometer, and isometric handheld dynamometer. The unit of measurement was N-M.

### 2.5 Data synthesis and analysis

Since the purpose of this study was to compare the effects of different therapeutic exercise through NMA, the exercise intervention was considered the experimental group, while the control group could be no intervention or any exercise therapy that was different from the experimental group. When both experimental and control groups included therapeutic exercise, the original group allocation as reported in the primary studies was retained and presented in the table of included study characteristics. Accordingly, in this study, a NMA was conducted by incorporating various intervention modalities as nodal arms.

Specifically, NMA was performed to synthesize and compare pre- and post-intervention data on ankle inversion muscle strength. The pre-to-post changes in the experimental and control groups were pooled to estimate the effects. The calculation method of the standard deviation (SD) of the change was based on the formula provided by the Cochrane Handbook for Systematic Reviews of Interventions (version 6.3) (Higgins et al., 2019) (the formula is: ${\text{SD}}_{\text{change}}=\sqrt{{\text{SD}}_{\text{baseline}}^{2}+{\text{SD}}_{\text{final}}^{2}-\left(2\times \text{Correlation}\times {\text{SD}}_{\text{baseline}}\times {\text{SD}}_{\text{final}}\right)}$ ) (Higgins et al., 2019). NMA was conducted using Stata software. A random effects multivariate NMA was performed within a frequentist framework (Salanti, 2012). Standardized mean differences were employed to pool effect sizes for outcomes with heterogeneous units. Weighted mean differences were applied for continuous variables, with merged effect estimates reported alongside 95% confidence intervals (CIs) and 95% prediction intervals.

A visualization tool was used for the graphical representation of the network geometry. The network diagram comprised nodes and edges, symbolizing training modalities and direct comparisons, respectively. Node weights corresponded to the number of participants allocated to each intervention, while edge thickness reflected the quantity of studies evaluating specific pairwise comparisons. For every outcome, the role of the direct and indirect evidence in the network summary was determined. Following the calculation of all pairwise summary effects and variances, a weighted least squares method was implemented to estimate contributions within the network contribution plot.

Inconsistency was evaluated at global and local levels (Hutton et al., 2015). Global inconsistency was assessed using a Wald test within a fitted inconsistency model, while local inconsistency was examined through loop inconsistency tests (a loop-specific approach using the method of moments) and node-splitting methods. In any other case, the inconsistent results were obtained. Heterogeneity standard deviations were quantified for all pairwise comparisons. If a P value more than 0.5 in the test of inconsistency indicated a statistically insignificant difference, the test of consistency would be conducted.

In addition, forest plots were constructed to visualize pooled effect sizes of direct pairwise comparisons across included interventions. Table 1 illustrates the complete matrix of results from the NMA providing the relative effectiveness for each pair of comparison. Prediction interval plots (pairwise comparison forest plots) were generated by computing effect sizes under both random- and fixed-effects models, enabling visual assessment of consistency between intervention pairs.

**TABLE 1:** Characteristics of the included studies.

Studies	Participants				Intervention			Outcome	
	N (M/F)	Age (year) (Mean ± SD)	Height (cm) (Mean ± SD)	Weight (kg) (Mean ± SD)	Experimental group	Control group	Frequency	Duration	Measurements method
Alizamani et al., 2023	36 (0/36)	Group A: 22.5 ± 2.63 Group B: 22.1 ± 2.76 Control group: 22.6 ± 2.91	Group A: 170.8 ± 3.91 Group B: 167.6 ± 2.83 Control group: 168.9 ± 3.84	Group A: 67.4 ± 2.87 Group B: 68.6 ± 2.36 Control group: 69.4 ± 3.02	Group A (unstable surface): core stability training on the trampoline Group B (stable surface): core stability training on the ground The core stability training was used a 3-kg medicine ball	The subjects received no intervention	3 sessions of 30–50 min per week	8 weeks	The isokinetic Biodex system III (Biodex medical systems, Shirely, NY)
Bagherian et al., 2019	42 (42/0)	Experimental group: 21.2 ± 1.7 Control group: 20.9 ± 1.8	Experimental group: 174.5 ± 6.1 Control group: 178.2 ± 6.6	Experimental group: 69.6 ± 6.9 Control group: 68.8 ± 8.1	Isometric and eccentric contraction strength training, as well as functional exercise	The subjects received no intervention	3 sessions of 60 min per week	8 weeks	The Biodex isokinetic dynamometer
Chang et al., 2021	63 (0/63)	Group A: 20.31 ± 1.28 Group B: 20.43 ± 1.25 Control group: 21.23 ± 1.47	Group A: 168.34 ± 5.78 Group B: 166.8 ± 6.84 Control group: 169.53 ± 4.78	Group A: 61.01 ± 22.39 Group B: 58.83 ± 13.14 Control group: 58.67 ± 16.54	Group A: Participants performed the exercises while standing on the vibration platform Group B: Participants performed the exercises on the BOSU balance ball. Both groups were asked to maintain balance on either leg or an affected leg while having eyes closed	The subjects received no intervention	3 sessions per week	6 weeks	The System 3 PRO dynamometer
Hall et al., 2015	39 (17/22)	Experimental group: 19.7 ± 2.2 Control group: 20.5 ± 2.1	Experimental group: 172.9 ± 12.8 Control group: 175.2 ± 8.1	Experimental group: 69.1 ± 13.5 Control group: 70.2 ± 11.1	Participants sat on the floor with 1 end of the band wrapped around a treatment table and the other end around the metatarsal heads of the involved foot. With the participants in a long sitting position, they were asked to perform resistance exercises in four directions: ankle dorsiflexion, plantar flexion, inversion, and eversion. The band was stretched to an additional 70% of its resting length to allow for consistent resistance tension among participants	The subjects received no intervention	3 sessions per week	6 weeks	An isometric handheld dynamometer (Manual Muscle testing system; Lafayette instruments co, Lafayette, IN)
Park et al., 2024	51 (51/0)	Group A: 14.1 ± 0.7 Group B: 14.2 ± 0.8 Control group: 14.4 ± 0.7	Group A: 167.6 ± 8.7 Group B: 169.9 ± 7.2 Control group: 170.2 ± 9.3	Group A: 53.9 ± 7.8 Group B: 55.4 ± 7.3 Control group: 57.6 ± 9.6	Group A (strength training): While seated, the participants wrapped a thera-band around the metatarsal heads of the involved foot, and performed three-directional isotonic ankle strength exercises: dorsiflexion, eversion, and inversion. For ankle plantarflexion, the participants performed a single-leg heel raise over the full ROM Group B (neuromuscular training): The protocol consisted of a single-leg stance, a single-leg stance while swinging the raised leg, a single-leg squat (30°–40°), and a single-leg stance while performing functional activity (catching or kicking). Both groups only involved the affected leg	The subjects received no intervention	3 sessions per week	6 weeks	A handheld dynamometer (K-Push®, KINVENT, montpellier, France)
Park et al., 2021	25	Experimental group: 22.58 ± 2.31 Control group: 21.69 ± 1.65	Experimental group: 167.28 ± 8.61 Control group: 165.68 ± 8.88	Experimental group: 58.27 ± 8.18 Control group: 57.63 ± 7.07	The strength training program included eccentric, concentric and isometric contractions exercise	The program consisted of a progressive form of exercise with the eyes open and closed conditions, including one-leg standing on a stable surface, one-leg standing on an unstable surface, maintaining normal posture, and maintaining posture while throwing a medicine ball	3 sessions of 20 min per week	3 weeks	A handheld dynamometer (Commander Muscle Tester, JTECH medical Inc., United States)
Samakosh et al., 2022	36 (36/0)	Experimental group: 21.08 ± 1.78 Control group: 20.58 ± 1.37	Experimental group: 177.58 ± 7.15 Control group: 178.58 ± 2.88	Experimental group: 76.41 ± 9.21 Control group: 71.83 ± 7.60	The protocol consisted balance training in stable and unstable planes and core stability strength training	The subjects received no intervention	3 sessions of 45–60 min per week	8 weeks	The isometric dynamometer (North Coast medical Inc, CA, United States)
Shamseddini Sofla et al., 2021	34 (21/13)	Experimental group: 40.58 ± 8.76 Control group: 38.40 ± 10.49	Experimental group: 163.91 ± 8.53 Control group: 166.20 ± 9.50	Experimental group: 74.88 ± 11.48 Control group: 73.60 ± 9.10	The participants received the whole-body vibration while they wore the shoe with an unstable surface	The subjects received no intervention between the initial and final assessments	3 sessions per week	4 weeks	An isokinetic dynamometer
Smith et al., 2012	40 (20/20)	Experimental group: 20.9 ± 2.2 Control group: 20.2 ± 2.1	Experimental group: 173.0 ± 7.9 Control group: 173.7 ± 8.2	Experimental group: 76.4 ± 16.1 Control group: 78.8 ± 24.5	Part 1: The participant sat on the floor with one end of the tubing tied around a treatment table and the other end around the metatarsal heads of the involved foot. The knees were fully extended, and Thera-Band was stretched to 170% of its resting length Part 2: Participants seated on the multiaxial Ankle Exerciser with their lower legs hanging over the edge of the table. The involved foot was secured onto the foot plate the thigh was parallel to the floor, promoting primary force generation by the lower leg Exercises in both parts were performed in 4 directions: dorsiflexion, plantar flexion, inversion, and eversion	The subjects received no intervention	3 sessions per week	6 weeks	A self-made isometric muscle strength testing device

The surface under the cumulative ranking (SUCRA) probability was used to rank the efficacy of different exercise therapies, and the coefficients and 95% CIs were used to evaluate the effect of each of interventions compared to a control. SUCRA values, ranging from 0 (lowest therapeutic certainty) to 100 (highest therapeutic certainty), were calculated to rank intervention efficacy. Elevated SUCRA values indicated superior therapeutic performance.

Finally, to check whether NMA publication bias caused by small-scale studies occurred, we generated a network funnel plot and conducted an intuitive check using the symmetry criterion.

## 3 Results

### 3.1 Literature search and trial selection

**FIGURE 1:**
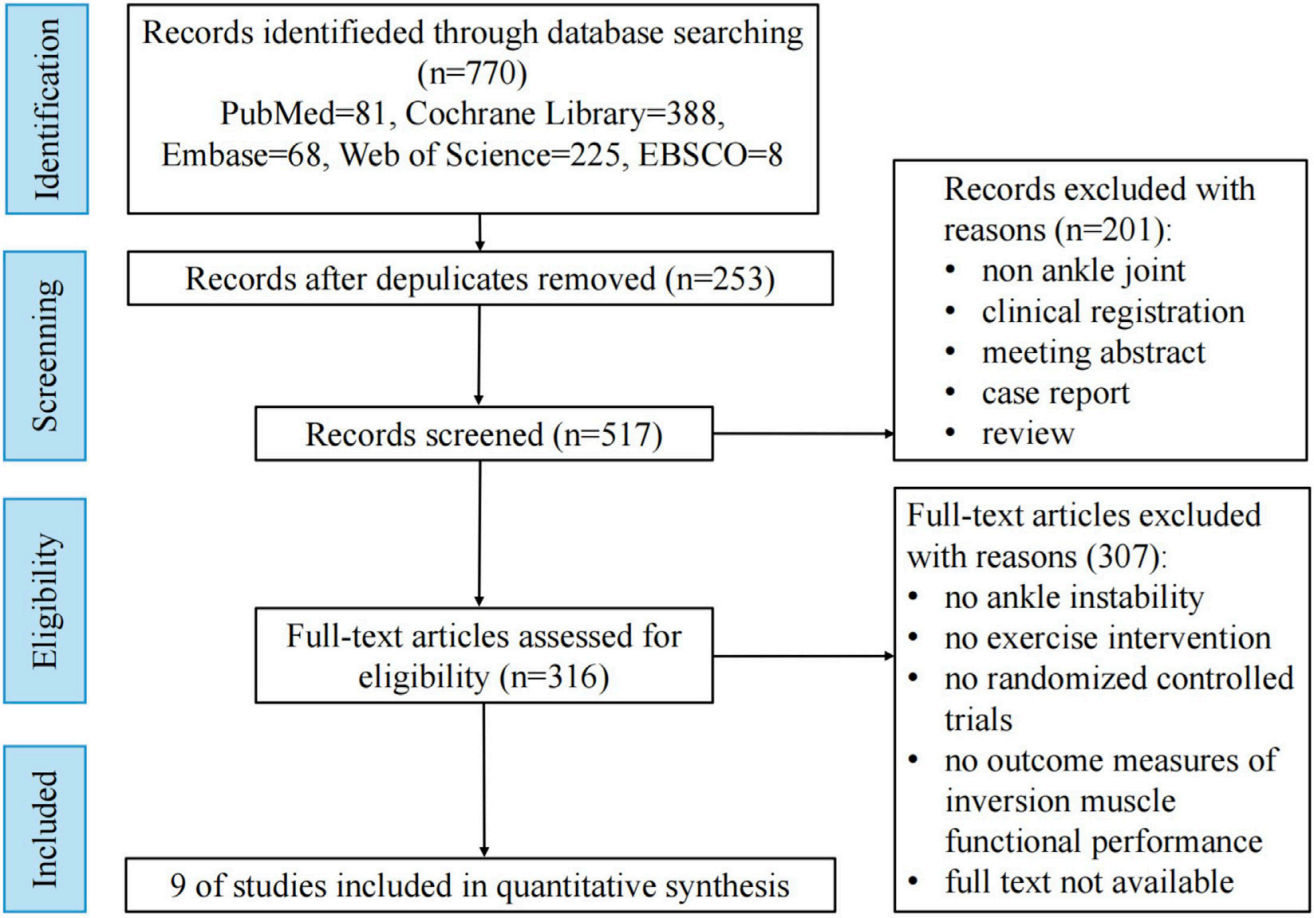
Flowchart of study selection.

A total of 770 potentially relevant articles were initially identified from the databases. Following the exclusion of duplicates, 201 records were removed during title and abstract screening due to irrelevance to the research topic. Subsequent full-text evaluation led to the exclusion of an additional 307 articles, resulting in the final inclusion of nine studies for NMA. Details of the selection process are illustrated in Figure 1.

### 3.2 Description of the included trials

The analysis encompassed 366 participants. Among the included studies, three exclusively recruited male participants, two enrolled female participants only, and three involved mixed-sex cohorts, while one study did not report gender-specific data. Interventions across studies were predominantly administered over 6–8 weeks, with a consistent frequency of three sessions per week. To standardize comparisons, exercise therapy modalities were classified according to internationally recognized consensus criteria (de Vries et al., 2011; Cruz et al., 2019). For studies employing multimodal rehabilitation protocols, only the primary intervention component was retained for analysis. The finalized intervention categories included NT, strength training (ST), combined neuromuscular and strength training (NST), and combined neuromuscular and whole-body vibration training (NWBVT).

The therapeutic interventions analysed included eight studies included a control group (no training), 3 for NT group (Chang et al., 2021; Park et al., 2024; Park et al., 2021), 5 for ST group (Alizamani et al., 2023; Hall et al., 2015; Park et al., 2024; Park et al., 2021; Smith et al., 2012), 3 for NST group (Alizamani et al., 2023; Bagherian et al., 2019; Mohammadi Nia Samakosh et al., 2022), and 2 for NWBVT group (Chang et al., 2021; Shamseddini Sofla et al., 2021). In addition, outcome measurement methodologies varied: five studies utilized automated isokinetic dynamometers, three employed manual isokinetic dynamometers, and one implemented a self-designed isometric muscle strength testing device. All measurement tools were validated as appropriate for assessing ankle strength in CAI populations. Comprehensive characteristics of the included studies are summarized in Table 1.

### 3.3 Methodological quality assessment

The methodological quality assessment of included studies is summarized in Table 2. Risk of bias was assessed with Cochrane Collaboration Bias Risk Assessment Tool (Sterne et al., 2019). Overall, six studies were rated as low risk and three as moderate risk. Four studies exhibited unclear risk in the randomization process, while most studies lacked blinding during post-intervention outcome assessments. One study demonstrated high risk due to substantial participant attrition, potentially compromising data completeness (Shamseddini Sofla et al., 2021). Overall, the quality of the included literature was relatively high.

**TABLE 2:** Methodological quality assessment of included studies.

Study	Allocation generation	Concealnent of allocation	Blinding of outcome assessment	Dropout rate %	Selective reporting	Other bias	Risk category
Alizamani et al., 2023	Unclear	Unclear	Unclear	17	Low	Low	Low
Bagherian et al., 2019	Low	Low	Unclear	13	Low	Low	Low
Chang et al., 2021	Low	Low	Unclear	0	Low	Unclear	Low
Hall et al., 2015	Unclear	Unclear	Unclear	13	Low	Low	Low
Park et al., 2024	Low	Low	Low	0	Low	Low	Low
Park et al., 2021	Unclear	Unclear	Unclear	Not reported	Low	Low	Moderate
Samakosh et al., 2022	Low	Low	Unclear	8	Low	Unclear	Low
Shamseddini Sofla et al., 2021	Low	Low	Low	24	Low	Low	Moderate
Smith et al., 2012	Unclear	Unclear	Unclear	Not reported	Low	Low	Moderate

### 3.4 Synthesis of the results

**FIGURE 2:**
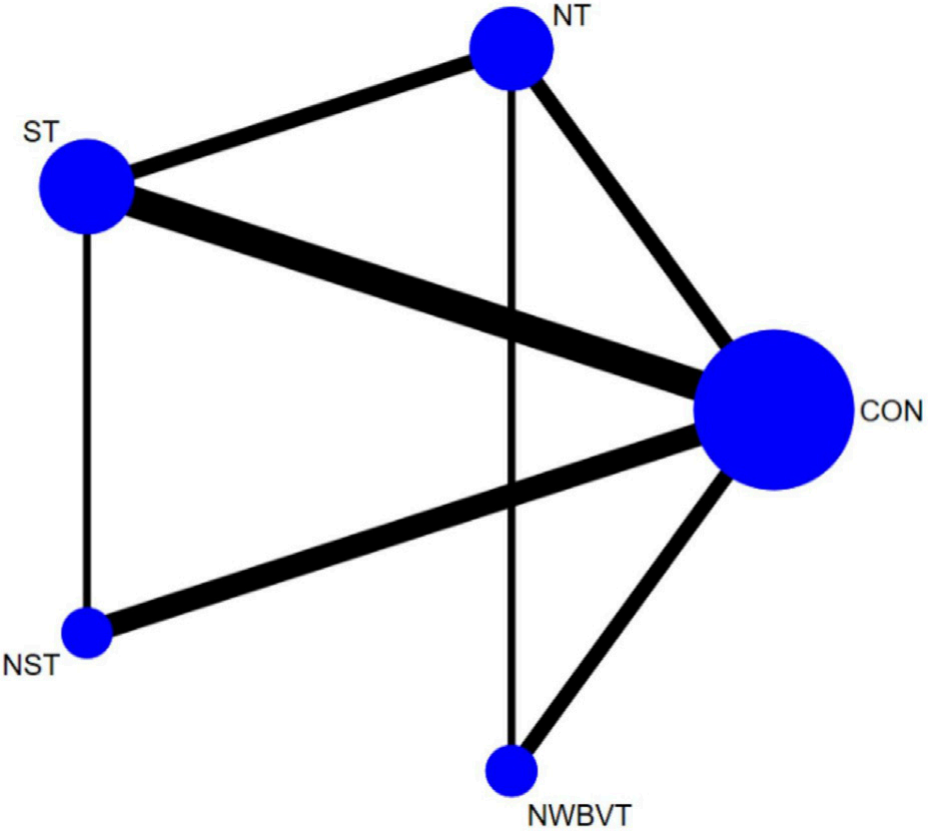
Network meta-analysis plot for the assessment of exercise therapy. The weight of each node is determined by the number of trials including the corresponding treatments. The larger the node and the thicker the line, the more studies are involved. Abbreviation: CON, control group; NT, neuromuscular training; ST, strength training; NST, combined neuromuscular and strength training; NWBVT, combined neuromuscular and whole-body vibration training.

To compare the efficacy of exercise interventions on ankle inversion function, a network comprising five interventions (including a control group) was constructed. After excluding 124 participants in the non-exercise control group, the remaining intervention groups included neuromuscular training (NT, n = 51), strength training (ST, n = 74), combined neuromuscular and strength training (NST, n = 42), and combined neuromuscular and whole-body vibration training (NWBVT, n = 33). A network contribution plot was generated to visualize the proportional influence of direct comparisons within the mixed-treatment network, with detailed contributions quantified in Figure 2.

Loop inconsistency tests for merged effect sizes yielded non-significant results (p > 0.05), indicating negligible heterogeneity and robust consistency across studies. Global and local inconsistency models (assessed via Wald tests and node-splitting methods) further confirmed no statistically significant discrepancies between direct and indirect comparisons (p > 0.05), reinforcing the reliability of the NMA findings.

A forest plot was generated to visualize heterogeneity across included studies (Supplementary Material). Subsequently, consistency models were applied following the absence of significant inconsistency. All interventions (NT (p = 0.007); ST (p = 0.00)); NST (p = 0.000)) demonstrated statistically significant improvements compared to the control group, except for NWBVT (p = 0.052) which demonstrated marginal significance. Direct and indirect evidence alignment was observed across comparisons, with inconsistency factors approximating zero, indicative of high methodological coherence.

**FIGURE 3:**
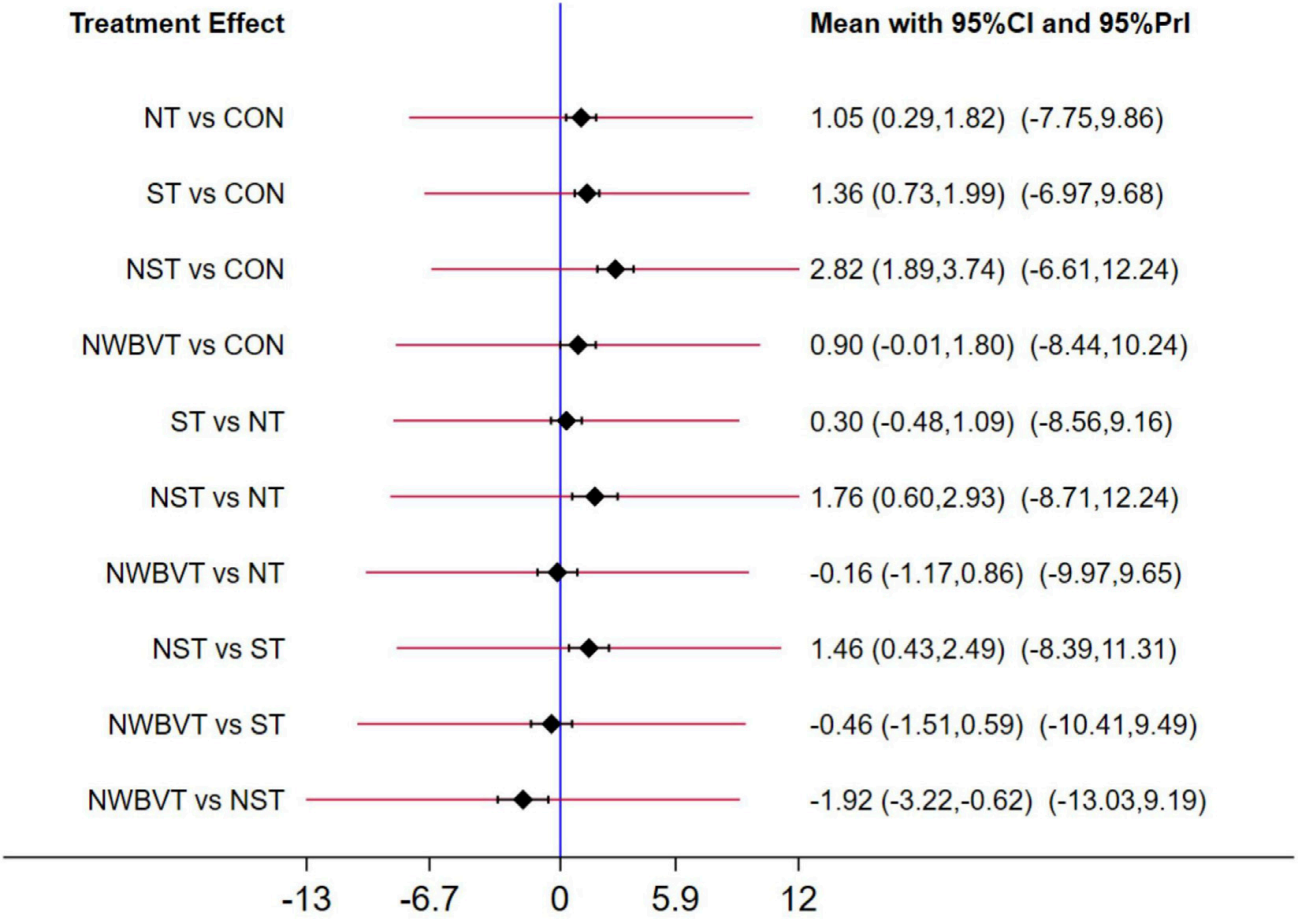
Prediction interval plots for the assessment of exercise therapy. Abbreviation: CON, control group; NT, neuromuscular training; ST, strength training; NST, combined neuromuscular and strength training; NWBVT, combined neuromuscular and whole-body vibration training.

**FIGURE 4:**
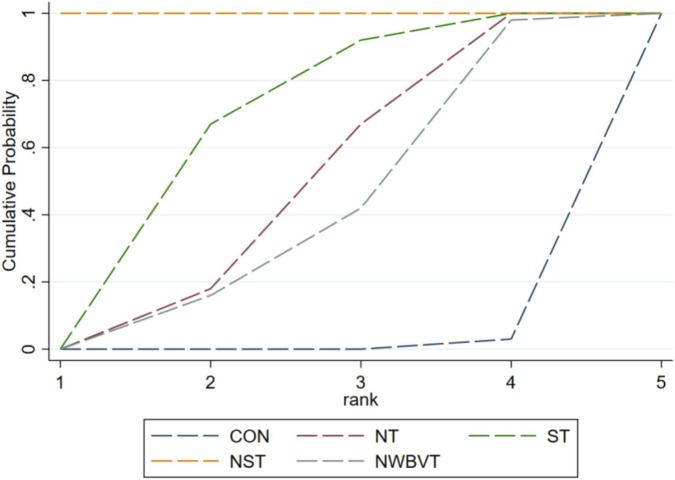
The complete matrix for the assessment of exercise therapy. Abbreviation: CON, control group; NT, neuromuscular training; ST, strength training; NST, combined neuromuscular and strength training; NWBVT, combined neuromuscular and whole-body vibration training.

Prediction interval plots (Figure 3) revealed consistency in all pairwise comparisons except NWBVT vs. NST, which exhibited differences. The complete matrix of results was showed in Figure 4. Overall, all therapeutic exercise modalities demonstrated statistically significant improvements in efficacy compared to control conditions (NT (Effect size (ES) = 1.05; 95%CI = 0.29–1.82), ST (ES = 1.36; 95%CI = 0.73–1.99), NST (ES = 2.82; 95%CI = 1.89–3.74), NWBVT (ES = 1.92; 95%CI = 0.62–3.22)). Among combined interventions, NST exhibited superior efficacy compared to NWBVT, and emerged as the most effective intervention overall.

**FIGURE 5:**
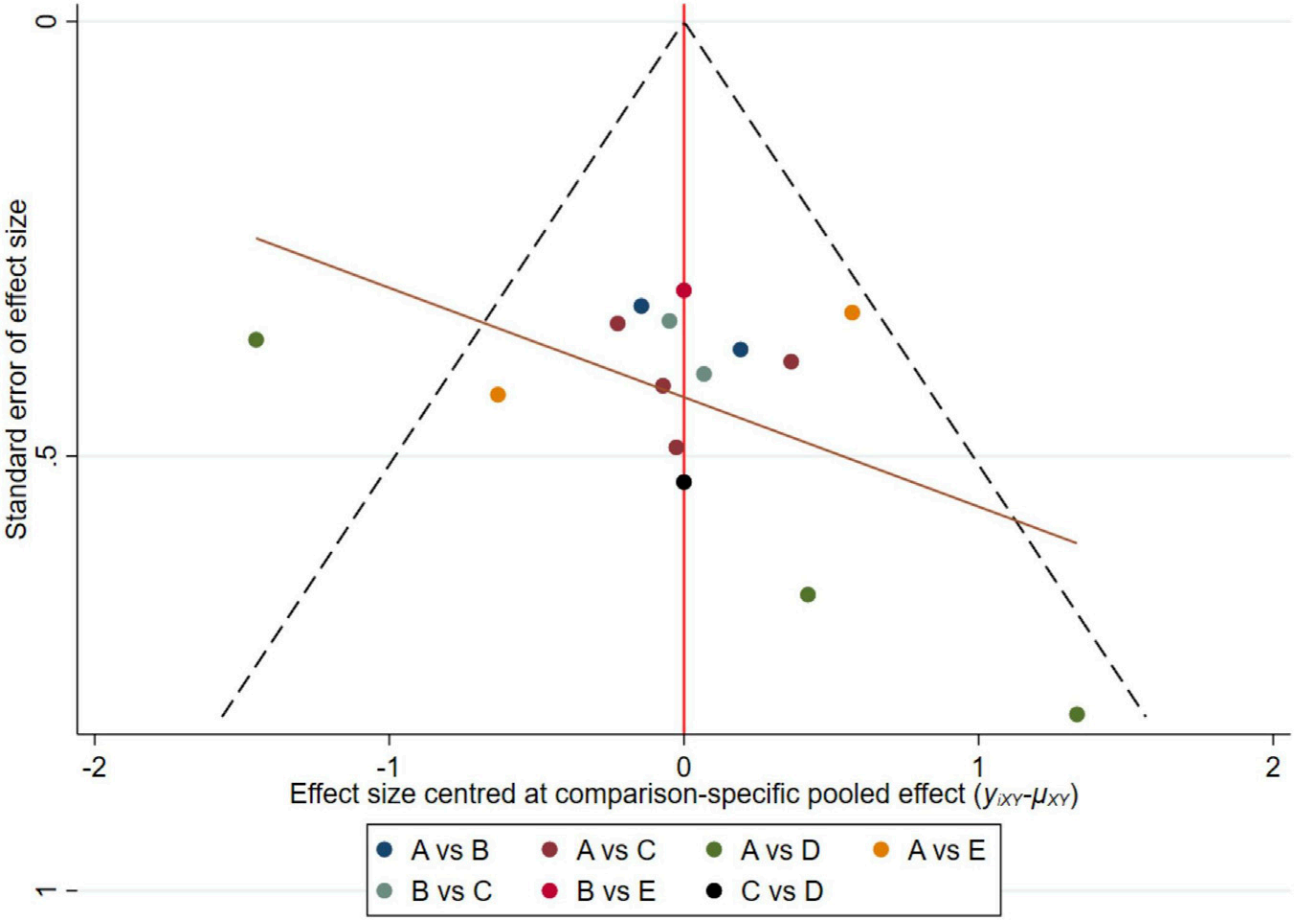
Surface under the cumulative ranking analysis for the assessment of exercise therapy. Abbreviation: CON, control group; NT, neuromuscular training; ST, strength training; NST, combined neuromuscular and strength training; NWBVT, combined neuromuscular and whole-body vibration training.

SUCRA analysis identified NST as the highest-ranked intervention (99.9%) (Figure 5). ST alone demonstrated a significant therapeutic advantage over NT. In contrast, NWBVT yielded the lowest therapeutic probability (38.9%). In general, all exercise-based interventions showed statistically significant differences in efficacy compared to the control group (0.7%).

### 3.5 Evolution of the publication bias

**FIGURE 6:**
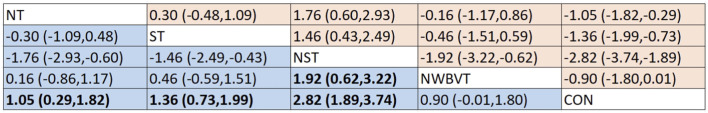
Funnel plot analysis for the assessment of exercise therapy. CON, control group. NT, neuromuscular training. ST, strength training. NST, combined neuromuscular and strength training. NWBVT, combined neuromuscular and whole-body vibration training.

Publication bias was evaluated through funnel plot analysis, which demonstrated approximate symmetry of included studies relative to the central axis, indicating no substantial evidence of bias (Figure 6). This symmetry reflects robust methodological quality across the synthesized literature.

## 4 Discussion

Our study is the first systematic review and NMA investigating the efficacy of exercise interventions on ankle inversion muscle functional performance in CAI. The findings revealed that all therapeutic exercise modalities were statistically and clinically effective in improving ankle inversion muscle functional performance. The effect sizes derived indicate superior efficacy of all exercise interventions relative to control conditions, except for NWBVT. Specifically, NST group demonstrated the most pronounced therapeutic effects, followed by ST and NT groups, compared to non-exercise control groups. Prediction interval plots identified inconsistency solely in the NWBVT vs. NST comparison, likely attributable to variabilities in effect estimation introduced due to the limited studies involving NWBVT. NST emerged as the optimal intervention (SUCRA: 99.9%), demonstrating superior specificity and efficacy compared to isolated NT or ST. The superiority of NST over isolated ST may be explained by the incorporation of neuromuscular training, which creates destabilizing conditions to enhance neuromuscular regulation (Alizamani et al., 2023). External perturbations in unstable environments stimulate proprioceptive feedback via muscle spindles and Golgi tendon organs, optimizing motor unit recruitment efficiency and reactivating atrophied periarticular musculature in CAI individuals (Mohammadi Nia Samakosh et al., 2022). Neuromuscular training improves neuromuscular control ability by exposing individuals to controlled instability, forcing the nervous system to refine motor strategies (Guo et al., 2024). Simultaneously, foot and ankle muscle strengthening exercise may have a good effect when used to enhance sensorimotor control in individuals with CAI (Han et al., 2022a). The repeated application of loading through the incorporation of resistance training has been shown to enhance neuromuscular adaptations, thereby facilitating an increase in muscle spindle sensitivity (Docherty et al., 1998). Therefore, this combination training was observed to accelerate functional ankle recovery and motor learning, helping individuals regain normal movement patterns. These associations emphasis the need for clinicians and rehabilitation specialists to prioritize integrated strength and neuromuscular training protocols in CAI rehabilitation, as this combined approach represents the most effective strategy for symptom improvement and functional restoration.

ST ranked second in therapeutic probability (64.6%). ST directly enhances muscle strength through mechanical tension-induced satellite cell differentiation and hypertrophy of type II muscle fibers (Dumont et al., 2015). Thera-band or manual resistance training can directly target inversion muscle groups through controlled antagonistic loading, inducing selective contraction of atrophied fibers (Hall et al., 2015). Using different training methods such as isometric and isotonic strength training further enhances neuromuscular recruitment and force generation (Park et al., 2021). However, biomechanical analyses further suggest a functional interdependence between ankle joint mechanics and proximal kinetic chain components (Friel et al., 2006). The core, as the central hub of force transmission, critically influences joint kinematics and myodynamic responses in the extremities (Ellenbecker and Davies, 2001; Marzieh et al., 2012). Enhanced core function facilitates improved neuromuscular control around the ankle joint through the modulation of force transduction pathways, thereby promoting more efficient muscle activation patterns during dynamic tasks (Kulas et al., 2008). Current studies have identified reduced core stabilizer strength in individuals with lower limb injuries relative to uninjured controls. Evidence indicates that core muscle strengthening interventions optimize stress distribution across the lower extremities, mitigating excessive mechanical loading on periarticular ankle structures (Sharma et al., 2011). However, only one study here included core training intervention, so further studies need to be conducted for determination of efficacy (Alizamani et al., 2023). Moreover, a systematic review and meta-analysis indicated that ST did not produce any minimal detectable changes on SEBT and Ankle Ability Measure scores in individuals with CAI (Luan et al., 2021). Overall, we suggest prioritized ST to address baseline muscular deficits when external constraints or individual-specific limitations prevent NST implementation.

Notably, NT alone ranked third in therapeutic efficacy (45.9%). Previous studies have emphasized the critical role of NT in restoring joint stability in CAI individuals (McKeon et al., 2008). Interestingly, our results indicate that NT protocols involving unstable surfaces significantly enhance inversion function during the constant-speed test despite differing fundamentally from strength training in mechanistic focus (Hiller et al., 2011). This beneficial effect may arise from the induced activation of perimalleolar neuromuscular pathways, where sustained proprioceptive challenges promote adaptive increases in muscle activation thresholds and ligamentous tolerance. Further, neuromuscular training increases the sensory stimulation required between the skin and the joints, and also increases the neuromuscular reactions caused by the stimulation of the body’s placement mechanism. Such adaptations likely facilitate the restoration of normative movement patterns in CAI individuals, consistent with existing mechanistic evidence (Tsikopoulos et al., 2018). Another study explained that the benefits of training on an unstable surface include increasing muscle function due to proper mobilization of motor units and neuromuscular activity timing (Remple et al., 2001). NT increases the cross-sectional area of muscles that maintain body stability and improves neuromuscular coordination (Anderson and Behm, 2005). However, while NT improves distal joint function indirectly via core stabilization, its focus on postural control probably limits direct strength gains compared to ST. NT primarily activates atrophied musculature post-injury and induces localized strength adaptations, which may insufficiently address systemic periarticular functional deficits. Upon achieving matched functional performance thresholds, NT should be incorporated as an adjunct modality to augment neuromuscular control capacity.

NWBVT demonstrated the lowest therapeutic probability (38.9%) in the intervention ranking. Whole body vibration (WBV), a training modality characterized by low mechanical load and high safety, is widely utilized in therapeutic exercise regimens. By generating mechanical oscillations at specific frequencies and amplitudes via vibration platforms, WBV activates primary endings of muscle spindles, stimulates joint mechanoreceptors, and enhances excitability of motor neurons, thereby modulating cortical activity and promoting motor unit recruitment to adapt to external stimuli (Sierra-Guzmán et al., 2018; Cloak et al., 2013; Moezy et al., 2008). Critically, WBV-induced sensory stimulation activates α-motor neurons, triggering muscle contractions that improve neuromuscular control deficits in CAI individuals (Aminian Far et al., 2016). In application, NWBVT relies on externally generated neuro-biomechanical feedback, potentially reducing individuals’ autonomy in achieving therapeutic goals. Participants may become overly dependent on external stimuli, inadvertently neglecting the development of intrinsic neuromuscular recruitment capacity. These findings emphasis the need for further high-quality studies to determine the value of WBV-based interventions.

This NMA study has several limitations. First of all, heterogeneity in demographic characteristics (e.g., age, gender, occupation) among participants may introduce outcome variability (Shao et al., 2022). Secondly, analytical bias may arise from inconsistencies in the types and protocols of testing instruments utilized for outcome measurement. The task-specific characteristics of different inversion muscle training protocols may account for the discrepancies observed in clinical outcome measure and self-reported function (Han et al., 2022b). For instance, variability in intervention protocols and implementation details across combined exercise therapies may influence treatment effect estimation. In addition, the analysis focused on broad classifications of exercise therapies without granular evaluation of the submodalities. For example, Alizamani et al. (2023) only carried out core strength training alone during training protocols. Regarding studies of neuromuscular training, Park et al. (2021) implemented progressive training protocols involving stable-to-unstable surfaces and eyes-open-to-closed transitions. Such methodological heterogeneity within intervention categories may obscure differential effects of specific training parameters. Subsequent research should stratify interventions by biomechanical focus (e.g., proximal vs. distal strength training) and complexity progression of training to explore optimal therapeutic strategies. Moreover, only two studies investigating NWBVT were included (Shamseddini Sofla et al., 2021; Chang et al., 2021), limiting the statistical power and generalizability of conclusions regarding this intervention. Future investigations should incorporate additional WBV-related studies to strengthen evidence synthesis. Moreover, other potential factors, such as abnormal processing of ankle proprioceptive information, may act synergistically as contributing mechanisms to invertor muscle dysfunction (Han et al., 2021; Han et al., 2015; Witchalls et al., 2012). Central nervous system integrative dysfunction may have a significant effect on peripheral joint kinematics and biomechanics.(Xu et al., 2025). Further clinical research is required to verify these hypotheses.

## 5 Conclusion

NMA demonstrated that combined neuromuscular and strength training may exhibit the most effective therapeutic exercise modality for enhancing inversion muscle function in individuals with CAI. This finding suggests that interventions incorporating ankle periarticular muscular strengthening and neuromuscular regulation yield superior therapeutic outcomes compared to isolated training approaches.

## Data Availability

The original contributions presented in the study are included in the article/Supplementary Material, further inquiries can be directed to the corresponding authors.
