# Supplementary material for: Which therapeutic exercise is most effective for improving ankle inversion muscle function in individuals with chronic ankle instability? A systematic review and network meta-analysis
Source: Front Bioeng Biotechnol. 2025 Dec 18;13:1691203. doi: 10.3389/fbioe.2025.1691203 (PMC12756897; doi:10.3389/fbioe.2025.1691203)
Supplement: Supplementary file 1 [file DataSheet1.docx]

Supplementary Material

## Supplementary Figures


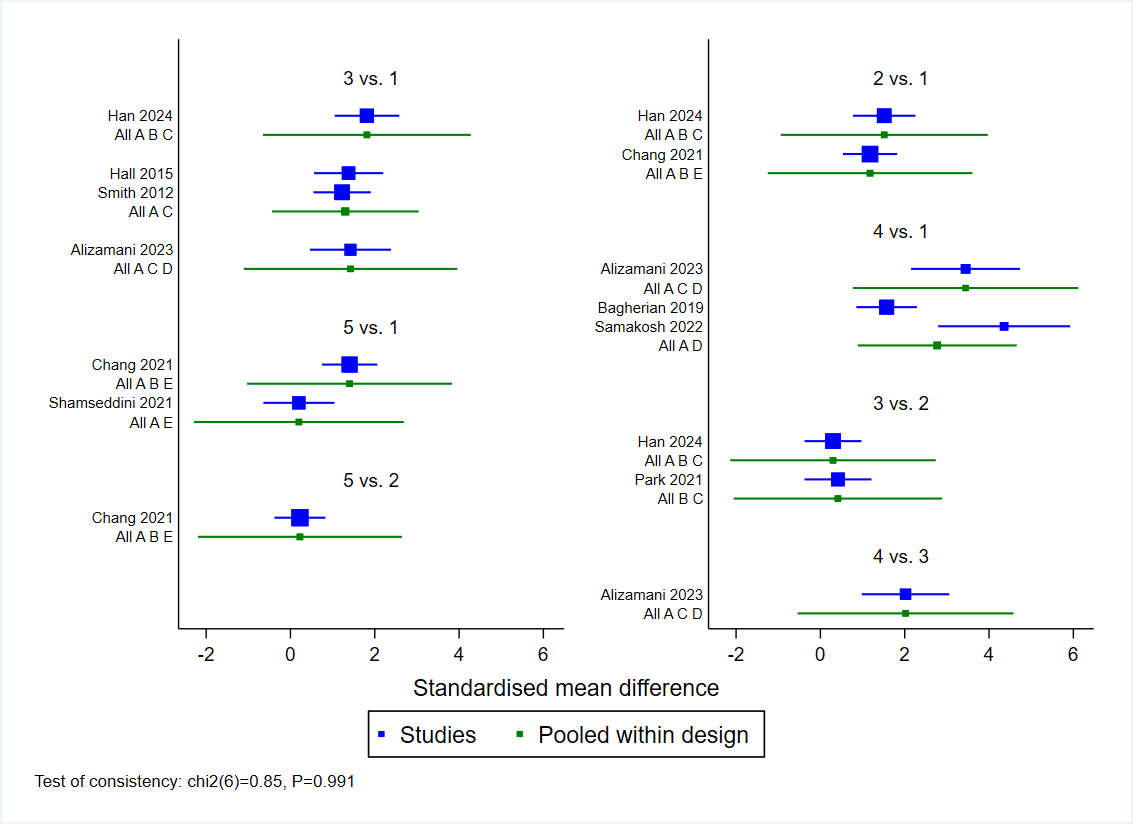


**Supplementary Figure 1.** **Forest plot for global inconsistency.** 1(A): control group. 2(B): neuromuscular training. 3(C): strength training. 4(D): combined neuromuscular and strength training. 5(E): combined neuromuscular and whole-body vibration training.

1. **Supplementary Tables**

| Study | Type | Mean | SD | N |
| --- | --- | --- | --- | --- |
| Alizamani 2023 | 4 | 8.06 | 2.162040703 | 10 |
| Alizamani 2023 | 3 | 2.84 | 2.700933172 | 10 |
| Alizamani 2023 | 1 | -0.84 | 2.636277679 | 10 |
| Bagherian 2017 | 4 | 5.7 | 4.490434277 | 20 |
| Bagherian 2017 | 1 | -1.3 | 4.237452065 | 20 |
| Chang 2021 | 5 | 3.8 | 6.042731171 | 21 |
| Chang 2021 | 2 | 2.39 | 5.589168096 | 21 |
| Chang 2021 | 1 | -4.99 | 6.864087703 | 21 |
| Hall 2015 | 3 | 45.7 | 28.94325483 | 15 |
| Hall 2015 | 1 | 0.7 | 34.46377809 | 14 |
| Han 2024 | 3 | 62.9 | 21.36773268 | 17 |
| Han 2024 | 2 | 55.5 | 20.72954413 | 17 |
| Han 2024 | 1 | 18.1 | 29.77384758 | 17 |
| Park 2020 | 3 | 0.05 | 0.072111026 | 12 |
| Park 2020 | 2 | 0.02 | 0.067082039 | 13 |
| Smith 2012 | 3 | 20.1 | 20.65177958 | 20 |
| Smith 2012 | 1 | -3 | 16.01124605 | 20 |
| Samakosh 2022 | 1 | 0.17 | 0.435361918 | 12 |
| Samakosh 2022 | 4 | 1.92 | 0.333256658 | 12 |
| Shamseddini 2021 | 5 | -0.32 | 6.011029862 | 12 |
| Shamseddini 2021 | 1 | -1.99 | 9.930181267 | 10 |

**Supplementary Tables1. Original dataset**

| chi2(6) | = | 0.85 |
| --- | --- | --- |
| Prob> chi2 | = | 0.9908 |

**Supplementary Tables2. Evaluation of global inconsistency using Wald test within a fitted inconsistency model**

| Loop | IF | seIF | z_value | p_value | CI_95 | Loop_Heterog_tau2 |
| --- | --- | --- | --- | --- | --- | --- |
| CON-BT-BWBVT | 0.791 | 0.555 | 1.426 | 0.154 | (0.00,1.88) | 0 |
| CON-ST-BST | 0.632 | 1.298 | 0.487 | 0.626 | (0.00,3.18) | 0.568 |
| CON-BT-ST | 0.352 | 0.43 | 0.817 | 0.414 | (0.00,1.20) | 0 |

**Supplementary Tables3. Evaluation of local inconsistency using loop-specific heterogeneity estimates. Abbreviation:** CON: control group. NT: neuromuscular training. ST: strength training. NST: combined neuromuscular and strength training. NWBVT: combined neuromuscular and whole-body vibration training.

| Side | Direct | | Indirect | | Difference | |  |
| --- | --- | --- | --- | --- | --- | --- | --- |
|  | Coef. | Std. Err. | Coef. | Std. Err. | Coef. | Std. Err. | P>\|z\| |
| A B | 1.332255 | 0.4652745 | 0.4382584 | 0.6957094 | 0.8939968 | 0.8409245 | 0.288 |
| A C | 1.444432 | 0.3708762 | 0.9127369 | 0.876493 | 0.5316949 | 0.959011 | 0.579 |
| A D* | 2.787836 | 0.5202637 | 3.436685 | 1.539658 | -0.648849 | 1.614707 | 0.688 |
| A E* | 0.8399703 | 0.5312588 | 1.393994 | 1.666575 | -0.5540238 | 1.752885 | 0.752 |
| B C | 0.3528596 | 0.5295651 | 0.204385 | 0.783739 | 0.1484746 | 0.9465002 | 0.875 |
| B E | 0.222202 | 0.6615982 | -0.8181594 | 0.8734236 | 1.040361 | 1.095215 | 0.342 |
| C D | 1.943367 | 0.7638455 | 1.09897 | 0.6809095 | 0.8443964 | 0.9985089 | 0.398 |

**Supplementary Tables4. Evaluation of local inconsistency using node-splitting methods.** A: control group. B: neuromuscular training. C: strength training. D: combined neuromuscular and strength training. E: combined neuromuscular and whole-body vibration training.

|  | Coef. | Std. Err. | z | P>z | [95% Conf. | Interval] |
| --- | --- | --- | --- | --- | --- | --- |
|  |  |  |  |  |  |  |
| _y_B |  |  |  |  |  |  |
| _cons | 1.054294 | 0.3921299 | 2.69 | 0.007 | 0.2857335 | 1.822854 |
|  |  |  |  |  |  |  |
| _y_C |  |  |  |  |  |  |
| _cons | 1.35779 | 0.3211153 | 4.23 | 0 | 0.7284154 | 1.987164 |
|  |  |  |  |  |  |  |
| _y_D |  |  |  |  |  |  |
| _cons | 2.816396 | 0.4735716 | 5.95 | 0 | 1.888213 | 3.744579 |
|  |  |  |  |  |  |  |
| _y_E |  |  |  |  |  |  |
| _cons | 0.8987821 | 0.4621784 | 1.94 | 0.052 | -0.0070709 | 1.804635 |

**Supplementary Tables5. Evaluation of consistency within a fitted consistency model. Abbreviation:** cons: control group. B: neuromuscular training. C: strength training. D: combined neuromuscular and strength training. E: combined neuromuscular and whole-body vibration training.

| Treatment | SUCRA | PrBest | MeanRank |
| --- | --- | --- | --- |
| BST | 99.9 | 99.5 | 1.0 |
| ST | 64.6 | 0.3 | 2.4 |
| BT | 45.9 | 0.1 | 3.2 |
| BWBVT | 38.9 | 0.1 | 3.4 |
| CON | 0.7 | 0.0 | 5.0 |

**Supplementary Tables6. The surface under the cumulative ranking (SUCRA) probability. Abbreviation:** CON: control group. NT: neuromuscular training. ST: strength training. NST: combined neuromuscular and strength training. NWBVT: combined neuromuscular and whole-body vibration training.
